# Supplementary material for: Large mammal burrows in late Miocene calcic paleosols from central Argentina: paleoenvironment, taphonomy and producers
Source: PeerJ. 2018 May 22;6:e4787. doi: 10.7717/peerj.4787 (PMC5969051; doi:10.7717/peerj.4787)
Supplement: Article S1 — Plot of average horizontal diameter of fossil burrows vs age of the hosting rock. Table containing raw data for Fig. 1 and literature source. Image credit: María Cristina Cardonatto and Ricardo Néstor Melchor. [file peerj-06-4787-s001.pdf]

Supplemental Article S1

Large mammal burrows in late Miocene calcic paleosols from central Argentina: palaeoenvironment, taphonomy and producers by M.C. Cardonatto and R.N. Melchor

Source of information for Figure 1

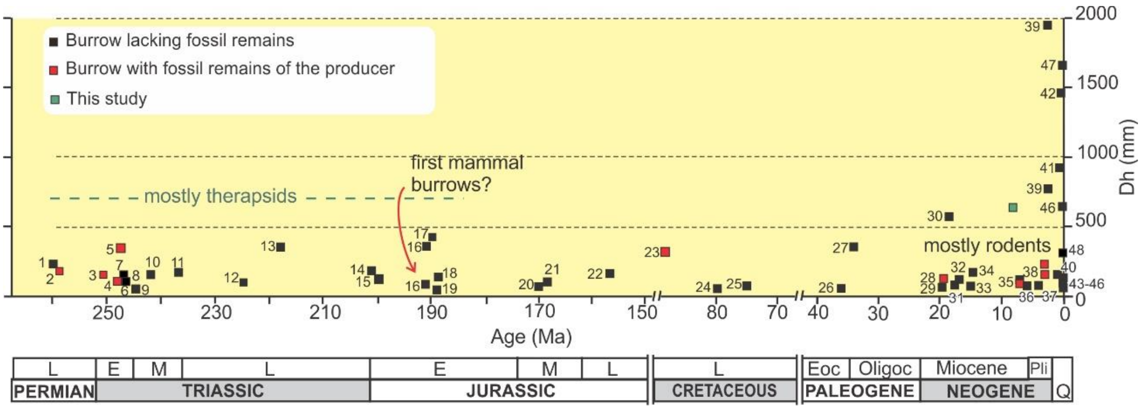

Plot of average horizontal diameter of fossil burrows vs age of the hosting rock.

Raw data for Figure 1

Symbol and source #	Age of hosting rock (Ma)	Average Dh (mm)	Fossil remains of producer
1	260	230	
2	258.5	160	X
3	251	157	X
4	248	122	X
5	247.5	340	X
6	247	121	
7	247	157	
8	247	126	
9	245	52	
10	242	155	
11	237	175	
12	225	100	
13	218	350	
14	201	180	
15	200	127	
16	191	93	
16	191	350	
17	190	420	
18	189	51	
19	189	146	

20	170	78	
21	169	95	
22	157	175	
23	96	320	X
24	80	50	
25	75	75	
26	36	44	
27	34	350	
28	19.5	125	X
29	19.5	65	
30	18.5	570	
31	17.5	85	
32	17	105	
33	15	66	
34	14.5	167	
This study	8	634	
35	7	90	
35	7	80	X
36	6	65	
37	4	77	
38	3	157	X
38	3	227	X
39	2.5	770	
39	2.5	1950	
40	1	150	
41	0.5	920	
42	0.4	1460	
43	0.04	89	
44	0.03	60	
45	0.03	80	
46	0.03	120	
47	0.015	1638	
48	0.015	605	

Source of data:

1. Liu J, Li L. 2013. Large tetrapod burrows from the Permian Naobaogou Formation of the Daqingshan Area, Nei Mongol, China. Acta Geologica Sinica - English Edition;87:1501-1507.
2. Smith RMH. 1987. Helical burrow casts of therapsid origin from the Beaufort Group (Permian) of South Africa. Palaeogeogr Palaeocl Palaeoecol;60:155–169.
3. Damiani R, Modesto S, Yates A, Neveling J. 2003. Earliest evidence of cynodont burrowing. Proceedings of the Royal Society of London B: Biological Sciences;270:1747–1751.

4. Groenewald GH, Welman J, MacEachern JA. 2001. Vertebrate burrow complexes from the Early Triassic *Cynognathus* Zone (Driekoppen Formation, Beaufort Group) of the Karoo Basin, South Africa. Palaios;16:148–160.
5. Modesto SP, Botha-Brink J. 2010. A burrow cast with *Lystrosaurus* skeletal remains from the Lower Triassic of South Africa. Palaios;25:274–281.
6. Krummeck WD, Bordy EM. 2017. *Reniformichnus katikatii* (New Ichnogenus and Ichnospecies): Continental Vertebrate Burrows from the Lower Triassic, Main Karoo Basin, South Africa. Ichnos:1-12.
7. Sidor CA, Miller MF, Isbell JL. 2008. Tetrapod burrows from the Triassic of Antarctica. J Vertebr Paleontol;28:277–284.
8. Miller MF, Hasiotis ST, Babcock LE, Isbell JL, Collinson JW. 2001. Tetrapod and large burrows of uncertain origin in Triassic high paleolatitude floodplain deposits, Antarctica. Palaios;16:218–232.
9. Sidor CA, Miller MF, Isbell JL. 2008. Tetrapod burrows from the Triassic of Antarctica. J Vertebr Paleontol;28:277–284.
10. Voigt S, Schneider JW, Saber H, Hminna A, Lagnaoui A, Klein H, Brosig A, Fischer JAN. 2011. Complex tetrapod burrows from the Middle Triassic red beds of the Argana Basin (Western High Atlas, Morocco). Palaios;26:555–566.
11. Fiorelli LE, Rocher S, Martinelli AG, Ezcurra MD, Hechenleitner EM, Ezpeleta M. 2018. Tetrapod burrows from the Middle–Upper Triassic Chañares Formation (La Rioja, Argentina) and its palaeoecological implications. Palaeogeogr Palaeocl Palaeoecol. (in press).
12. Colombi CE, Jofré C, Currie BS. 2008. Large-diameter burrows in the Upper Triassic Ischigualasto Formation, northwestern Argentina. Ameghiniana;45:797–801.
13. Colombi CE, Fernández E, Currie BS, Alcober OA, Martínez R, Correa G. 2012. Large-diameter burrows of the Triassic Ischigualasto Basin, NW Argentina: Paleoecological and paleoenvironmental implications. PLoS ONE;7:e50662.
14. Bordy EM, Sciscio L, Abdala F, McPhee BW, Choiniere JN. 2017. First Lower Jurassic vertebrate burrow from southern Africa (upper Elliot Formation, Karoo Basin, South Africa). Palaeogeogr Palaeocl Palaeoecol;468:362-372.
15. Csiki-Sava, Z., Kędzior, A., Pieńkowski, G., Popa, M.E., 2016. Hettangian tetrapod burrows from the continental Steierdorf Formation at Anina, western Romania. Geological Quarterly, 60: 395–406.
16. Riese DJ, Hasiotis ST, Odier GP. 2011. Synapsid Burrows and Associated Trace Fossils in the Lower Jurassic Navajo Sandstone, Southeastern Utah, U.S.A., Indicates a Diverse Community Living in a Wet Desert Ecosystem. J Sediment Res;81:299–325.
17. Loope DB. 2006. Burrows dug by large vertebrates into rain-moistened Middle Jurassic sand dunes. Journal of Geology;114:753–762.
18. Melchor RN, Loope D. 2016. Communal therapsid burrows from interdune facies of the Lower Jurassic Navajo Sandstone, southern Utah, USA. Abstract book Ichnia2016: p.174.
19. Engelmann GF, Chure DJ, Good TR. 2014. Large burrows in the dunes of the Nugget Sandstone, Early Jurassic?, of NE Utah. New Mexico Museum of Natural History and Science, Bulletin;62:197-203.
20. Xing L, Peng G, Klein H, Ye Y, Jiang S, Burns ME, Ran H. 2017. Middle Jurassic tetrapod burrows preserved in association with the large sauropod Omeisaurus jiaoi from the Sichuan Basin, China. Historical Biology;29:931-936.

21. Loope DB. 2008. Life beneath the surfaces of active Jurassic dunes: Burrows from the Entrada Sandstone of south-central Utah. Palaios;23:411–419.
22. Dentzien-Dias PC, Schultz CL, Bertoni-Machado C. 2008. Taphonomy and paleoecology inferences of vertebrate ichnofossils from Guará Formation (Upper Jurassic), southern Brazil. Journal of South American Earth Sciences;25:196-202.
23. Varricchio DJ, Martin AJ, Katsura Y. 2007. First trace and body fossil evidence of a burrowing, denning dinosaur. Proceedings of the Royal Society B: Biological Sciences;274:1361–1368.
24. Simpson EL, Hilbert-Wolf HL, Wizevich MC, Tindall SE, Fasinski BR, Storm LP, Needle MD. 2010. Predatory digging behavior by dinosaurs. Geology;38:699-702.
25. Martin AJ, Varricchio DJ. 2011. Paleoecological utility of insect trace fossils in dinosaur nesting sites of the Two Medicine Formation (Campanian), Choteau, Montana. Historical Biology;23:15–25.
26. Melchor RN, Bedatou E, de Valais S, Genise JF. 2006. Lithofacies distribution of invertebrate and vertebrate trace-fossil assemblages in an Early Mesozoic ephemeral fluvio-lacustrine system from Argentina: Implications for the *Scoyenia* ichnofacies. Palaeogeogr Palaeocl Palaeoecol;239:253–285.
27. Bellosi ES, Laza JH, Sánchez MV, Genise JF. 2010. Ichnofacies analysis of the Sarmiento Formation (middle Eocene – early Miocene) at Gran Barranca, Central Patagonia. In: RH Madden, Carlini AA, Vucetich MG, Kay RF, The Paleontology of Gran Barranca Evolution and Environmental Change through the Middle Cenozoic of Patagonia. p. 302–312.
28. Martin LD, Bennett DK. 1977. The burrows of the Miocene beaver Palaeocastor, western Nebraska, U.S.A. Palaeogeogr Palaeocl Palaeoecol;22:173–193.
29. Gobetz KE, Martin LD. 2006. Burrows of a gopher-like rodent, possibly *Gregorymys* (Geomyoidea: Geomyidae: Entoptychtinae), from the early Miocene Harrison Formation, Nebraska. Palaeogeogr Palaeocl Palaeoecol;237:305–314.
30. Hunt RMJ, Xiang-Xu XUE, Kaufman J. 1983. Miocene burrows of extinct bear dogs: Indication of early denning behavior of large mammalian carnivores. Science;221:364–366.
31. Gee CT, Sander PM, Petzelberger BEM. 2003. A Miocene rodent nut cache in coastal dunes of the Lower Rhine Embayment, Germany. Palaeontology;46:1133–1149.
32. Krapovickas V. 2012. Ichnology of distal overbank deposits of the Santa Cruz Formation (late Early Miocene): paleohydrologic and paleoclimatic significance. In: MS Bargo, Kay RF, Vizcaíno SF, Early Miocene Paleobiology in Patagonia: High-Latitude Paleocommunities of the Santa Cruz Formation. p. 91-103. Cambridge.
33. Melchor RN, Umazano AM, Perez M, Krause JM. 2016. Endemic bioturbation: distinctive Neogene eolian trace fossil assemblages dominated by large meniscate burrows from Patagonia, Argentina. Ichnia 2016, Fourth International Congress on Ichnology, Abstract Book, p. 208-209.
34. Gobetz KE. 2006. Possible burrows of mylagaulids (Rodentia: Aplodontoidea: Mylagaulidae) from the late Miocene (Barstovian) Pawnee Creek Formation of northeastern Colorado. Palaeogeogr Palaeocl Palaeoecol;237:119–136.

35. Joeckel RM, Tucker ST. 2013. Exceptionally well preserved latest Miocene (Hemphillian) rodent burrows from the eastern Great Plains, United States, and a review of the burrows of the North American rodents. Palaios; 28:793-824. / Voorhies MR. 1975. A New Genus and Species of Fossil Kangaroo Rat and Its Burrow. Journal of Mammalogy; 56:160-176.
36. Melchor RN, Genise JF, Umazano AM, Superina M. 2012. Pink fairy armadillo meniscate burrows and ichnofabrics from Miocene and Holocene interdune deposits of Argentina: Palaeoenvironmental and palaeoecological significance. Palaeogeogr Palaeocl Palaeoecol;350–352:149–170.
37. Morgan GS, Lucas SG. 2000. Pliocene and Pleistocene vertebrate faunas from Albuquerque Basin, New Mexico. New Mexico Museum of Natural History and Science Bulletin;16:217–240.
38. Genise JF. 1989. Las cuevas con *Actenomys* (Rodentia, Octodontidae) de la Formación Chapadmalal (Plioceno superior) de Mar del Plata y Miramar (Provincia de Buenos Aires). Ameghiniana;26:33–42. / Quintana CA. 1992. Estructura interna de una paleocueva, posiblemente de un Dasypodidae (Mammalia, Edentata), del Pleistoceno de Mar del Plata (Provincia de Buenos Aires, Argentina). Ameghiniana;29:87-91. / Scognamillo DG. 1993. Estructura de las cuevas de *Actenomys* (Rodentia: Octodontidae) de la Aloformación Playa San Carlos, Plioceno tardío (Barranca de los Lobos, Pdo. Gral. Pueyrredón): significado paleoecológico y estratigráfico. Unpublished Licenciate thesis, National University of Mar del Plata, Argentina, 32 pp. / Fernandez ME, Vassallo AI, Zárate M. 2000. Functional morphology and paleobiology of the pliocene rodent *Actenomys* (Caviomorpha: Octodontidae): the evolution to a subterranean mode of life. Biological Journal of the Linnean Society;71:71–90. / Elissamburu A, Dondas A, De Santis L. 2011. Morfometría de las paleocuevas de la "Fm." Capadmalal y su asignación a *Actenomys* (Rodentia), *Paedotherium* (Notoungulata) y otros mamíferos fósiles hospedantes. Mastozoologia Neotropical;18:227-238.
39. Quintana CA. 1992. Estructura interna de una paleocueva, posiblemente de un Dasypodidae (Mammalia, Edentata), del Pleistoceno de Mar del Plata (Provincia de Buenos Aires, Argentina). Ameghiniana;29:87-91. / Zárate MA, Bargo MS, Vizcaíno SF, Dondas A, Scaglia O. 1998. Estructuras biogénicas en el Cenozoico tardío de Mar del Plata (Argentina) atribuibles a grandes mamíferos. , 5(2),. Revista de la Asociación Argentina de Sedimentología;5:95-103. / Vizcaíno SF., Zárate M., Bargo MS, Dondas A. 2001. Pleistocene burrows in the Mar del Plata area (Argentina) and their probable builders. Acta Palaeontologica Polonica 46:289-301. / Dondas A, Isla FI, Carballido JL. 2009. Paleocaves exhumed from the Miramar Formation (Ensenadan Stage-age, Pleistocene), Mar del Plata, Argentina. Quaternary International;210:44-50.
40. Wood HE, Wood AE. 1933. *Daemonhelix* in the Pleistocene of Texas. The Journal of Geology;41:824-833.
41. Imbellone P, Teruggi M. 1988. Sedimentación crotovínica en secuencias cuaternarias bonaerenses. 1° Reunión Argentina de Sedimentología, Actas: 125-129.
42. Buchmann FS, Pereira Lopez R, Caron F. 2009. Icnofósseis (paleotocas e crotovinas) atribuídos a mamíferos extintos no sudeste e sul do Brasil. Revista brasileira paleontológica. 12 (3): 247-256. / Lopes RP, Frank HT, Buchmann FSdC, Caron F. 2017. *Megaichnus* igen. nov.: Giant Paleoburrows Attributed to Extinct Cenozoic Mammals from South America. Ichnos;24:133-145.

43. Lim HS, Lee YI, Yi S, Kim CB, Chung CH, Lee HJ, Choi JH. 2007. Vertebrate burrows in late Pleistocene paleosols at Korean Palaeolithic sites and their significance as a stratigraphic marker. Quaternary Research;68:213–219.
44. Tobin RJ. 2004. Ichnology of a late Pleistocene ichnofabric, Nebraska, USA. Palaeogeogr Palaeocl Palaeoecol;215:111–123.
45. Melchor RN, Genise JF, Umazano AM, Superina M. 2012. Pink fairy armadillo meniscate burrows and ichnofabrics from Miocene and Holocene interdune deposits of Argentina: Palaeoenvironmental and palaeoecological significance. Palaeogeogr Palaeocl Palaeoecol;350–352:149–170.
46. Schmeisser RL, Loope DB, Wedin DA. 2009. Clues to Medieval destabilization of the Nebraska Sand Hills, USA, from ancient pocket gopher burrows. Palaios;24:809–817.
47. Tauber AA. 2006. Crotovinas del Pleistoceno.Holoceno de Córdoba, Argentina. III Congreso Argentono de Cuaternario y Geomorfología, Actas: 711-716.
48. Tomassini RL, Montalvo CI, Beilinson E, Deschamps CM, Garrone MC, Gasparini GM, Zárate MA, Rabassa J, Ruella A, Tonni EP. 2017. Microvertebrates preserved in mammal burrows from the Holocene of the Argentine Pampas: a taphonomic and paleoecological approach. Historical Biology;29:63-75.
