# Supplementary material for: Large mammal burrows in late Miocene calcic paleosols from central Argentina: paleoenvironment, taphonomy and producers
Source: PeerJ. 2018 May 22;6:e4787. doi: 10.7717/peerj.4787 (PMC5969051; doi:10.7717/peerj.4787)
Supplement: Article S3 — Systematic assignation of bone remains found inside burrow fills. Credit: María Cristina Cardonatto. [file peerj-06-4787-s003.pdf]

Supplemental Article 3

Large mammal burrows in late Miocene calcic paleosols from central Argentina: palaeoenvironment, taphonomy and producers by M.C. Cardonatto and R.N. Melchor

Systematic paleontology

Subclass Eutheria Huxley, 1880
Superorder XENARTHRA Cope, 1889
Order Tardigrada Latham and Davies in Forster, 1795
Family Mylodontidae Gill, 1872
Subfamily Scelidotheriinae Ameghino, 1904
Genus *Proscelidodon* Bordas, 1935
Proscelidodon sp.
Fig. S1A-B

Material: Associated elements from SG (# 665). GHUNLPam 18807-1, right mandibular fragment with m_{1}-$_{2}$ and m_4 (Fig. S1A); GHUNLPam 18807-2, distal part of metacarpal II (Fig. S1B); GHUNLPam 18807-3 proximal part of metacarpal II; GHUNLPam 18807-4, carpal fragment; GHUNLPam 18807-5, fragmented radius distal epiphysis with articular facets; GHUNLPam 18807-6, posterior part of basicranium.

Description: In the mandibular fragment, the m_{1-2} are elongated antero-posteriorly, m_4 is bilobed and larger than the remaining teeth. Fragmentary metapodial remains corresponding to metacarpal II, including a distal portion with articular facets for the phalange and a proximal portion with articular facets with the carpal bones. The radius epiphysis fragment exhibits a concave facet that match the carpal remain.

Comments: The association of the postcranial elements with the mandible remain, and a consistent size of both suggest the assignation of the postcranial elements to *Proscelidodon* sp. Within the Cerro Azul Formation, *Elassotherium altirostre* (Rovereto, 1914) was mentioned by Cabrera (1939) from two nearby localities of the Buenos Aires province: Epecuén lake and Laguna La Paraguaya; whereas Zetti (1972) recorded this species from SG, La Pampa province. This taxon was considered a junior subjective synonym of *Proscelidodon almagroi* (Rovereto) by McDonald (1987) including the holotype of *E. altirrostre* from Laguna La Paraguaya. This author mentioned two species of Scelidotherinae in the Huayquerian SALMA: *P. gracillimus* at SG, Guaminí, Epecuén lake and Las Huayquerias de San Carlos (Mendoza province); and *P. almagroi* from Laguna La Paraguaya and Andalhualá (Catamarca province).
Molar configuration of the available material is typical of *Proscelidodon* sp, but a more detailed assignation is not possible.

Order Cingulata Illiger, 1811
Superfamily Glyptodontoidea Gray, 1869
Family Glyptodontidae Gray, 1869
Subfamily Hoplophorinae Huxley, 1864
Tribe Hoplophorini Huxley, 1864 (= Sclerocalyptini Ameghino, 1895)
Genus *Eosclerocalyptus* Ameghino, 1919
Eosclerocalyptus sp.
Fig. S1D

Material: MGH-P126/ 34, a portion of carapace composed of four articulated and fragmented osteoderms associated to four broken osteoderms from Laguna La Paraguaya (# 702).

Description: All osteoderms show a central circular to oval figure fringed by small peripheral figures bounded by marked grooves.

Comments: The surface ornamentation allows assignation to *Eosclerocalyptus* sp. The genus *Hoplophractus* was synonymized under *Eosclerocalyptus* by Zurita and Tomassini (2006). The holotype of

Hoplophractus tapinocephalus Cabrera, 1939 was collected at Laguna La Paraguaya locality, and this species was also identified at SG (Zetti, 1972) and Laguna Chillhué (Montalvo et al., 1995).

Glyptodontidae indet.
Fig. S1C, E, F

Material: GHUNLPam 18817 five isolated osteoderms from SG (# 632A); and MGH-P126/38, two articulated osteoderms plus eight isolated osteoderms, including two incomplete (# 687); MGH-P126/37 three isolated osteoderms (# 688) (Fig. S1E, F), and MGH-P126/41 a carapace fragment with several osteoderms and two isolated ones (# 670) (Fig. S1C), the three later from Laguna La Paraguaya.
Comments: The poor preservation of this material precludes a more detailed assignation.

Superfamily Dasypodoidea Gray, 1821
Family Dasypodidae Gray, 1821
Subfamily Euphractinae Pocock, 1924
Tribe Eutatini Bordas, 1933
Genus *Doellotatus* Bordas, 1932
Doellotatus sp.
Fig. S1G

Material: MGH-P126/40 a broken mobile osteoderm from Laguna La Paraguaya (# 720).
Description: Osteoderm with a wide central figure, surrounded by a shallow groove.
Comments: The first report of *Doellotatus* from La Pampa was by Bordas (1933), who described *D. chapadmalensis*; a species later reported by Esteban et al. (2001) and Urrutia et al. (2008) from SG. The last authors also recorded *D. inornatus* in SG. A more detailed taxonomic assignation is not possible because of the fragmentary state of the remain.

Order Notoungulata Roth, 1903
Suborder Typotheria Zittel, 1893
Family Hegetotheriidae Ameghino, 1894
Subfamily Pachyrukhinae Kraglievich, 1934
Genus *Paedotherium* Burmeister, 1888
Paedotherium minor Cabrera, 1937
Fig. S1H

Material: MGH-P126/39 a right mandibular fragment with a m_3 (# 698) and an uncollected tooth from the upper jaw included in a calcareous concretion (# 699) both from Laguna La Paraguaya.
Comments: *Paedotherium minor* is very common in the Cerro Azul Formation outcrops both from the eastern La Pampa province and western Buenos Aires province (Montalvo et al., 2016; Cerdeño et al., 2017).

Family Mesotheriidae Alston, 1876
Subfamily Mesotheriinae Simpson, 1945
Mesotheriinae indet.
Fig. S1I

Material: MGH-P126/42 a broken upper left incisor from Laguna La Paraguaya (# 689).
Description: The upper incisor exhibits a labial convexity and a subquadrangular section.
Comments: Cerdeño and Montalvo (2001) concluded that the only valid Mesotheriinae species for the late Miocene of central Argentina are: *Pseudotypotherium subinsigne* and *Typotheriopsis silveyrai*. The dental features that distinguish both species are scarce and they do not include any feature of the incisors, for this reason the described material can only be assigned to the subfamily level.

Undetermined mammal

Material: MGH-P126/35 axis, MGH-P126/36 a tibia fragment, two phalanges and a metapodial (# 701); MGH-P126/43 a cranial fragment (# 723) from Laguna La Paraguaya; and GHUNLPam 18808 a cranial remain and four metapodial fragments (# 660) from SG.

Comments: The fragmentary state of these fragments precludes a more detailed assignation.

References

Alston E R. 1876. On the classification of the Order Glires. Proceedings of the Zoological Society of London 1876: 61-98.

Ameghino F. 1894. Enumération synoptique des espèces de mammifères fossiles des formations éocènes de Patagonie: Boletín de la Academia Nacional de Ciencias en Córdoba. 13: 259-455.

Ameghino F. 1895. Sur les édentés fossiles de l´ Argentine (Examen critique, révision et correction de l` ouvrage de M. R. Lydekker "The extinct Edentates of Argentina"). Revista del Jardín Zoológico de Buenos Aires. 3: 97-192.

Ameghino F. 1904. Nuevas especies de mamíferos cretáceos y terciarios de la República Argentina. Anales de la Sociedad Científica Argentina. 58: 241-291.

Ameghino F. 1919. Sobre mamíferos fósiles del Piso Araucanense de Catamarca y Tucumán. I° Reunión Nacional de la Sociedad Argentina de Ciencias Naturales, Actas: 151-152 + Láminas III y VII.

Bordas AF. 1932. Proposición de nuevo género para *Eutatus inornatus*. Physis. 11: 167-168.

Bordas AF. 1933. Notas sobre los Eutatinae. Nueva Subfamilia extinguida de Dasypodidae. Anales del Museo Nacional de Historia Natural. 37: 583-614.

Bordas AF. 1935. Observaciones sobre los géneros *"Scelidodon"* Ameghino y *"Proscelidodon"* n. g. Physis. 11: 484-491.

Burmeister CV. 1888. Relación de un viaje a la Gobernación de Chubut. Anales del Museo Nacional de Buenos Aires. 3: 175-252.

Cabrera A. 1937. Notas sobre el suborden "Typotheria". Notas del Museo de La Plata 2: 17-43.

Cabrera A. 1939. Sobre vertebrados fósiles del Plioceno de Adolfo Alsina. Revista del Museo de La Plata (Nueva Serie). 2: 3-35.

Cerdeño E, Montalvo CI. 2001. Los Mesotheriinae (Mesotheriidae, Notoungulata) del Mioceno superior de La Pampa, Argentina. Revista Española de Paleontología. 16: 63-75.

Cerdeño E, Montalvo CI, Sostillo R. 2017. Deciduous dentition and eruption pattern in in late Miocene Pachyrukhinae (Hegetotheriidae, Notoungulata) from La Pampa province, Argentina. Historical Biology. 29 (3): 359-375.

Cope ED. 1889. The Edentata of North America. American Naturalist 23: 657-664.

Gill T. 1872. Arrangement of the families of mammals with analytical tables. Smithsonian Miscellaneous Collections 11: 1-98.

Esteban G, Nasif N, Montalvo CI. 2001. Nuevos registros de Dasypodidae (Xenarthra) del Mioceno tardío de la provincia de La Pampa (Argentina). Revista Española de Paleontología. 16: 77-87.

Gray JE. 1821. On the natural arrangement of vertebrate animals. London Medical Repository. 15: 296–310.

Gray J E. 1869. Catalogue of carnivorous, pachydermatous and edentate Mammalia in the British Museum. British Museum (Natural History), Londres. 398 p.

Huxley T.H. 1864. On the osteology of the genus *Glyptodon*. *Proceedings of the Royal Society of London* 13: 108.

Huxley TH. 1880. On the application of the laws of evolution to the arrangement of the Vertebrata, and more particularly of the Mammalia. Proceedings of the Zoological Society, London 43:649-662.

Illiger, C. 1811. Prodromus Systematis Mammalium et Avium Additis Terminis Zoographicis Utriusque Classis. Salfeld, Berlín. 301p.

Kraglievich L. 1934. La antigüedad pliocena de las faunas de Monte Hermoso y Chapadmalal, deducidas de su comparación con las que le precedieron y sucedieron. Imprenta "El Siglo Ilustrado", 136 p.

Latham J, Davies H. 1795. Faunula indica; appendix. In: Forster JR (Ed.), Zoologia Indica, 2nd edition. Editorial Secunda, Halle S. 38 p.

McDonald HG. 1987. A systematic review of the Plio-Pleistocene Scelidotherinae Ground Sloth (Mammalia: Xenarthra: Mylodontidae). PhD Tesis, University of Toronto, Toronto, Canada, 478 p. Unpublished.

Montalvo CI, Visconti G, Púgener L, Cardonatto MC. 1995. Mamíferos de Edad Huayqueriense (Mioceno tardío), Laguna Chillhué, provincia de La Pampa. 4° Jornadas Geológicas y Geofísicas Bonaerenses (Junín), Actas 1: 73-79.

Montalvo CI, Tomassini RL, Sostillo R. 2016. Leftover prey remains: a new taphonomic mode from the late Miocene (Cerro Azul Formation) in central Argentina. Lethaia. 49: 219-230.

Pocock RI. 1924. The external characters of the South American edentates. Procedures of the Zoological Society of London. 65: 983-1031.

Roth S. 1903. Noticias preliminares sobre nuevos mamíferos fósiles del Cretácico superior y Terciario inferior de la Patagonia: Revista del Museo de La Plata. 11: 133-158.

Rovereto, C. 1914. Los Estratos Araucanos y sus Fósiles. Anal. Mus.Nac. Hist. Nat. Buenos Aires, 25: 249 pp.

Simpson GG. 1945. The principles of classification and classification of mammals. Bulletin of the American Museum of Natural History. 85: 1-350.

Urrutia JJ, Montalvo CI, Scilato-Yané GJ. 2008. Dasypodidae (Xenarthra, Cingulata) de la Formación Cerro Azul (Mioceno tardío) de la provincia de La Pampa, Argentina. Ameghiniana. 45 (2): 289-302.

Zetti J. 1972. Los mamíferos fósiles de Edad Huayqueriense (Plioceno medio) de la región pampeana. Unpublished doctoral thesis. Facultad de Ciencias Naturales y Museo, Univesidad Nacional de La Plata.

Zittel KA. 1893. Handbuch der Palaeontologie, IV. Bd. Vertebrata (Mammalia): Munich, R. Oldenbourg, 590 p.

Zurita AE, Tomassini R. 2006. Revisión de un Hoplophorini poco conocido, *Sclerocalyptus lineatus* Ameghino (Mammalia, Glyptodontidae) de Edad Montehermense de la Argentina. Studia Geologica Salmatisencia. 42: 11-20.
