# Supplementary figures and images for: Large mammal burrows in late Miocene calcic paleosols from central Argentina: paleoenvironment, taphonomy and producers
Source: PeerJ. 2018 May 22;6:e4787. doi: 10.7717/peerj.4787 (PMC5969051; doi:10.7717/peerj.4787)

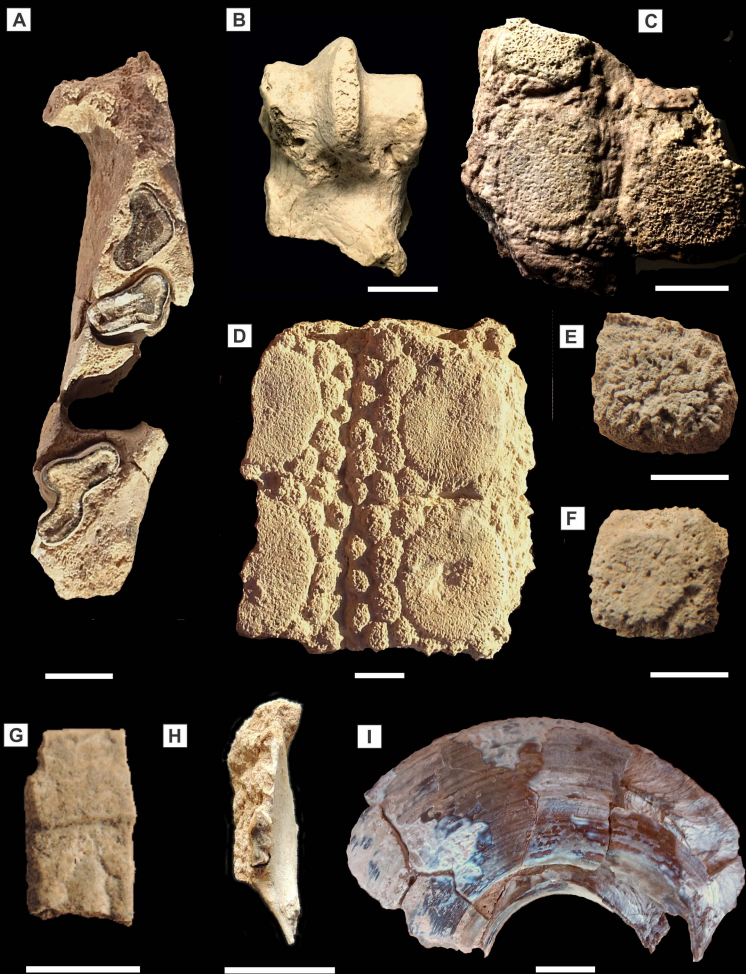

Supplement: Figure S1 — (A) Proscelidodon sp. (GHUNLPam 18807-1). (B) fragmented metacarpal of Proscelidodon sp. (GHUNLPam 18807-2). (C) Gliptodontidae indet. (MGH-P126/41). (D) Eosclerocalyptus sp. (MGH-P126/34). (E–F) Glyptodontidae indet. (MGH-P126/37). (G) Doellotatu s sp. (MGH-P126/40). (H) Paedotherium minor (MGH-P126/39). (I) Mesotheriinae indet. (MGH-P126/42). Scale bar: 1 cm. Image credit: María Cristina Cardonatto. [file peerj-06-4787-s004.pdf]
