# Supplementary material for: Large mammal burrows in late Miocene calcic paleosols from central Argentina: paleoenvironment, taphonomy and producers
Source: PeerJ. 2018 May 22;6:e4787. doi: 10.7717/peerj.4787 (PMC5969051; doi:10.7717/peerj.4787)
Supplement: Table S1 — References: SC: slightly curved, SI: sinuous, C: “C” shaped, E: elliptical, S: subcircular, PC: planoconvex, * approximate value, () minimum value. Dh, horizontal diameter; Dv, vertical diameter. Body mass estimation after Wu et al. (2015). Wu NC, Alton LA, Clemente CJ, Kearney MR, White CR. 2015. Morphology and burrowing energetics of semi-fossorial skinks (Liopholis spp.). The Journal of Experimental Biology, 218: 2416–2426. Credit: María Cristina Cardonatto. [file peerj-06-4787-s005.pdf]

Table S1. Burrow fills registered in the late Miocene Cerro Azul Formation. References: SC: slightly curved, SI: sinuous, C: "C" shaped, E: elliptical, S: subcircular, PC: planoconvex, * approximate value, () minimum value. Dh= horizontal diameter, Dv= vertical diameter. Body mass estimation after Wu et al. (2015).

burrow #	plan view pattern	cross-sectional shape	Dh (cm)	Dv (cm)	Dv/Dh	Cross section area (cm^2)	Body mass estimate (kg)	Bone remains inside burrow fill
**Salinas Grandes de Hidalgo** (SG, 37º 12' 55"S, 63º 35' 25"W)								
658	SC		20	(8)				
640		E	23	17	0.74			
647		E	39	20	0.51	1693.3	59.12	
632 B		S	40	37	0.92	2712.3	122.04	
662		E	40	20	0.5	1250.9	37.10	
622 A	SI		42					
635		E (PC)	46	28	0.61	2122.7	83.70	
657	SC		46	28	0.61			
637		S	54	57	1.05	2686.7	120.27	
629			*56	35				
665			*60	(30)				*Proscelidodon* sp.
661	SC		63	(35)				
622 B	SC		64					
623	SI		64					
646		E	64	38	0.59	3867.5	210.65	
661 A			65	(32)				
649		E	66	42	0.64	3939.5	216.72	
664	SC		68					
663		E	69	41	0.59			
611	SC		70					
645			70	(26)				
644		E (PC)	71	33	0.46	2999.6	142.49	
626	C		72					
631		E	72	41	0.57	6230.6	438.72	
648		E	73	39	0.53	3585.6	187.50	
659 A			74	(34)				
615	SC		75					
617	SC		75					
613 B	SC		76					
619	SC		78					
612	SC		79					
614	SC		80					
616	SC		80					
620	SC		80					
621	SC		80					
624 A	SI		80					
624 B	SC		83					
613 A	SC		85					

618	SC		87					
638		E	88	40	0.45	5692.4	381.79	
641		E	88	52	0.59			
625	SC		92					
633		E	98	47	0.48			
659		E	105	67	0.64	9278.1	809.53	
634		E	106	46	0.43			
639		E	106	60	0.57	8566.8	716.04	
643		E	114	60	0.53	9190.5	797.80	
642		E (PC)	125	92	0.74	12825.8	1332.23	
628		E	*126	50		8504.7	708.07	
630		E (PC)	128	80	0.62	14580.6	1622.77	
632 A		E	150	76	0.51	14183.6	1555.29	Glyptodontidae indet.
636				56				
660								Undetermined
**Laguna La Paraguaya** (LLP, 37° 5'53.57"S; 62°47'34.98"W)								
680 B	SC		15					
698	SC		27					*Paedotherium minor*
675	SC		28	(16)				
682	SC		28	(8)				
674	SC		30	(13)				
714	SC		32	(4)				
694	SC		34	(18)				
683		S	39	29	0.74	2270.6	92.84	
697	SC		40	(17)				
710	SC		40	(20)				
672	SC		42	(25)				
709	SC		42	(24)				
720	SC		43	(5)				*Doellotatus* sp.
686	C		44	(18)				
696	SC		44	(30)				
676	SC		45	(23)				
679	SC		45	(10)				
723	SC		46	(12)				Undetermined
701	SC		47					Undetermined
687	SC		50					Glyptodontidae indet.
688	SC		50	(38)				Glyptodontidae indet.
708	SC		50	(36)				
680 A	SC		52	(16)				
692	SC		52	(20)				
699	SC		52	(14)				*Paedotherium minor*
718		S	52	38	0,.73	2477.3	106.16	
703	C		54	(25)				
715	SC		54	35				
722		S	54	42	0.77	3205.4	157.80	
673	SC		55	(33)				
700	SC		55	(10)				
690	SC		56	(43)				
706	SC		56	(15)				
713		S	56	47	0.84	3566.8	185.99	

669	SC		58	(28)				
671	SC		43	(26)				
693	SC		58	(20)				
716	SC		58	54				
668	SC		60	(35)				
677	SC		60	(15)				
691	SC		60	(24)				
695	SI		60	(28)				
705	C		60	(18)				
711	SC		60	36				
712	SC		62	54				
719		E	64	27	0.42	5413.2	353.36	
717	SC		67	(9)				
685	C		68	(14)				
704	SC		68					
689	SC		70	(19)				Mesotheriinae indet.
678	SC		74	(27)				
702	SC		78					*Eosclerocalyptus* sp.
667	SC		79					
681	SC		80	(20)				
707	SC		85	(35)				
670	SC		94	(35)				Glyptodontidae indet.
721	SC		94	(14)				
666	SC		97	(32)				
684	SC		115	(40)				
**Laguna Chillhué** (LC, 37°19'15.13"S; 64°14'31.52"W)								
655		E	55	29	0.53	1263.3	37.67	
655 A		E	80	42	0.52	3045.2	145.83	
656			89	(25)				

Wu NC, Alton LA, Clemente CJ, Kearney MR, White CR. 2015. Morphology and burrowing energetics of semi-fossorial skinks (*Liopholis* spp.). The Journal of Experimental Biology, 218: 2416-2426.
